# Hydroxyapatite from Mollusk Shells: Characteristics, Production, and Potential Applications in Dentistry

**DOI:** 10.3390/dj12120409

**Published:** 2024-12-16

**Authors:** Florin Lucian Muntean, Iustin Olariu, Diana Marian, Teodora Olariu, Emanuela Lidia Petrescu, Tudor Olariu, George Andrei Drăghici

**Affiliations:** 1 Discipline of Surgery, “Victor Babeş” University of Medicine and Pharmacy, Eftimie Murgu Square No. 2, 300041 Timișoara, Romania; munteanu.florin@umft.ro; 2 Department of Dentistry, Faculty of Dentistry, “Vasile Goldiş” Western University of Arad, 94−96 Revoluţiei Blvd., 310025 Arad, Romania; marian.diana@uvvg.ro; 3 Department of Medicine, Faculty of Medicine, “Vasile Goldiş” Western University of Arad, Liviu Rebreanu No. 86, 310414 Arad, Romania; olariu.teodora@uvvg.ro; 4 Department of Prosthesis Technology and Dental Materials, Dental Research Center Using Conventional and Alternative Technologies, “Victor Babeş” University of Medicine and Pharmacy, Eftimie Murgu Square No. 2, 300041 Timișoara, Romania; petrescu.emanuela@umft.ro; 5 Department of Organic Chemistry, Faculty of Pharmacy, “Victor Babeș” University of Medicine and Pharmacy Timișoara, Eftimie Murgu Square No. 2, 300041 Timișoara, Romania; olariu.t@umft.ro; 6 Department of Toxicology, Faculty of Pharmacy, “Victor Babeș” University of Medicine and Pharmacy Timișoara, Eftimie Murgu Square No. 2, 300041 Timișoara, Romania; draghici.george-andrei@umft.ro; 7 Research Center for Pharmaco-Toxicological Evaluations, Faculty of Pharmacy, “Victor Babeș” University of Medicine and Pharmacy Timișoara, Eftimie Murgu Square No. 2, 300041 Timișoara, Romania

**Keywords:** mollusk shells, hydroxyapatite, dentistry, dental implants, bone grafts

## Abstract

Modern dentistry is turning towards natural sources to overcome the immunological, toxicological, aesthetic, and durability drawbacks of synthetic materials. Among the first biomaterials used as endosseous dental implants, mollusk shells also display unique features, such as high mechanical strength, superior toughness, hierarchical architecture, and layered, microporous structure. This review focusses on hydroxyapatite—a bioactive, osteoconductive, calcium-based material crucial for bone healing and regeneration. Mollusk-derived hydroxyapatite is widely available, cost-effective, sustainable, and a low-impact biomaterial. Thermal treatment coupled with wet chemical precipitation and hydrothermal synthesis are the most common methods used for its recovery since they provide efficiency, scalability, and the ability to produce highly crystalline and pure resulting materials. Several factors, such as temperature, pH, and sintering parameters, modulate the size, purity, and crystallinity of the final product. Experimental and clinical data support that mollusk shell-derived hydroxyapatite and its carbonated derivatives, especially their nanocrystaline forms, display notable bioactivity, osteoconductivity, and osteoinductivity without causing adverse immune reactions. These biomaterials are therefore highly relevant for specific dental applications, such as bone graft substitutes or dental implant coatings. However, continued research and clinical validation is needed to optimize the synthesis of mollusk shell-derived hydroxyapatite and determine its applicability to regenerative dentistry and beyond.

## 1. Introduction

Dental materials have undergone major advancements over the past decades, culminating with the development of composite resins, high-strength ceramics, and titanium-based dental implants [1]. Nonetheless, even such high-performance materials can encounter safety issues and adverse effects, including unfavorable immune reactions and mechanical wear problems [2]. Driven by the pressing need for improved durability, biocompatibility, functionality, and aesthetics, a major research focus of scientists working to overcome these challenges is put on biomaterials derived from natural sources [3]. Biomaterials with applicability in dentistry must fulfill specific functional requirements in terms of hardness, strength, and fracture toughness in order to ensure long-term reliability and performance in demanding applications [3]. In addition, they must be biocompatible and nontoxic, and ideally possess features like bioactivity, antifungal properties, and good aesthetic performance [4].

Natural materials display remarkable mechanical and biological attributes, often overcoming the performance of simple mixtures of their constituents or their artificial counterparts [1]. Natural calcium carbonate-based materials, such as seashells, corals, eggshells, or nacre, possess several key characteristics that make them suitable for various dental applications. Thus, calcium carbonate is biocompatible [5,6]—a feature that limits the risks of immune reactions (e.g., inflammation, rejection) and facilitates integration with bone tissues during the healing process [1,5]. Calcium carbonate is also abundant and inexpensive; therefore, these materials are cost-effective resources for use in the dental industry [2,3]. Functioning as a storage site for calcium ions, these biomaterials promote enamel remineralization and help reverse the initial stages of tooth decay [7]. Overall, these features make natural calcium carbonate-based materials valuable resources for modern dental practices.

Mollusk shells are renewable resources, being available in large quantities if natural populations are properly exploited and managed [8,9]. Their use leads to waste reduction by minimizing the need for mining and processing new calcium carbonate [10]. With a layered structure including both nanoscale and microscale layers, mollusk shells also share the same basic architecture as human bone [3]. These calcareous exoskeletons display a hierarchical structure, which is complemented by a textured surface and an intricate network of pores and crystalline arrangements. This architecture improves the mechanical strength of the shell and facilitates cell attachment/proliferation due to the increased surface area available for cell attachment [2,4,5]. Mollusk shell-derived materials are also promising alternatives for cosmetic dentistry since they can be tailored to mimic the visual characteristics of natural teeth, e.g., color, translucency, and surface texture [1].

Among mollusk shell-derived compounds, hydroxyapatite (HA) and chitin reveal excellent potential for use in dental applications [2,3,7,11,12]. The former compound exists naturally in bones and tooth enamel, but can be extracted from several other sources, including mollusk shells [1]. This form of calcium phosphate promotes bone growth and integrates well with bone tissue; hence, it is suitable for use in bone grafts and as coatings for dental and orthopedic implants [2]. In addition, HA is an in-demand material for scaffolds in bone tissue engineering and can be utilized in toothpaste to remineralize and strengthen enamel [3,5]. One of the most abundant natural polymers, chitin, is, by contrast, a polysaccharide composed of N-acetylglucosamine monomers, with a linear, chain-like structure [11]. It is mainly found in the exoskeletons of arthropods and, in much smaller amounts, in mollusk shells [1,11]. Typically used as chitosan, its deacetylated derivative, this biocompatible material is widely used in wound dressings and dental materials due to its antimicrobial, hemostatic, and healing-promoting properties [11]. Chitosan can also be used for coating dental and orthopedic implants, creating biocompatible scaffolds that promote cell attachment and proliferation, and targeted and controlled drug delivery [12].

Besides naturally obtained compounds, biopolymers can be synthetically produced to achieve specific characteristics for medical applications. High-profile examples of these compounds include polylactic-co-glycolic acid (PLGA) and polycaprolactone (PCL), which are commonly used in tissue regeneration and drug delivery systems and for bioresorbable membranes aiding tissue and bone regeneration, respectively [1,4]. It is, however, important to mention that, irrespective of their origin, natural/synthetic compounds used for medical and dental applications must meet the criteria of biocompetence, i.e., to interact effectively and harmoniously with biological systems, fulfilling specific roles without causing adverse reactions. This attribute encompasses several key features, including (i) biocompatibility: no adverse immune response or toxicity; (ii) bioactivity: actively supports cell growth and tissue healing; (iii) biodegradability: allows controlled breakdown, particularly useful in temporary scaffolds; (iv) mechanical integrity: provides necessary strength and durability; and (v) tailorability: customizable for specific functions, such as drug delivery or antimicrobial effects [1,2,11].

This review provides a comprehensive overview of the current use of mollusk shell-derived biomaterials in dentistry, focusing on hydroxyapatite (HA) and its carbonated derivatives. We discuss the architecture of mollusk shells, methods for obtaining HA, and key factors influencing these processes. Continuing with the current status of (pre)clinical studies on this topic and future directions of research, this review aims to broaden our knowledge and stimulate scientific interest in recovering HA from mollusk shells—an underexplored area of the larger puzzle of how natural biomaterials can be used for biomedical applications.

## 2. A Brief Overview of Mollusk Shell Architecture

**Figure 1:**
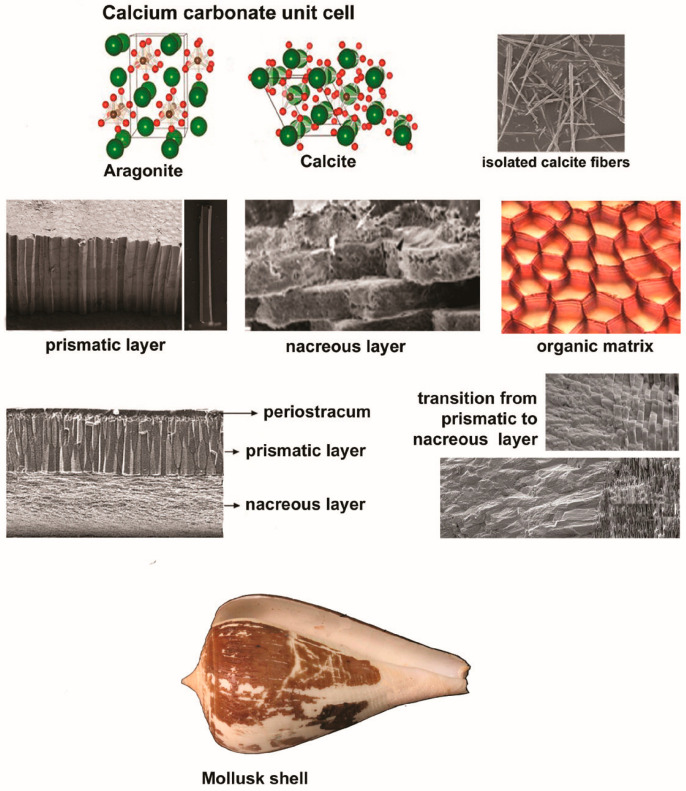
Structure of molluskan shells at nanoscale level (**first row**), microscale level (**second row**), mesoscale level (**third row**), and macroscale level (**fourth row**).

Most mollusks possess a hard shell with a protective role, except nudibrach gastropods, slugs, squids, octopuses, and cuttlefish, although the latter exhibits an internal shell, referred to as cuttlebone [13]. Mollusk shells present a highly organized hierarchical structure, with multiple levels of organization of both the organic and mineral components [13], as shown in Figure 1. From a chemical point of view, the molluskan shell is composed of calcium carbonate as aragonite, with a rhombohedral crystal structure, or calcite, with a more complex orthorhombic crystal structure. Aragonite is the predominant form of calcium carbonate for most mollusks, often being above 90% of the shell material [14]. The prevalence of aragonite over calcite is due to the biological and environmental conditions, primarily its superior stability [15], greater mechanical strength [16], and higher magnesium (Mg) to calcium (Ca) ratio in marine environments [17]. Besides organic macromolecules, molluskan shells contain trace elements, such as magnesium and strontium (Sr), which can influence their properties in terms of hardness, stability, and resistance to environmental stressors [14,18].

At the nanoscale level, fibrous microstructures composed of long, acicular calcium carbonate crystals fuse with granular microstructures consisting of spherical calcium carbonate crystals to create a robust yet flexible frame [19]. This structure interacts with an organic matrix comprising polysaccharides (e.g., chitin, glycosaminoglycans) and fibrous proteins (primarily nacrein), modulating the formation and growth of calcium carbonate crystals [14,19]. It is also important to mention that molluskan shells, irrespective of their taxonomic origin, always include few superimposed calcified layers (up to five), and an organic layer [20]. The outer, thin, organic, leathery layer, also known as the periostracum, plays an important role in protection and adhesion to substrates and, in some species, in osmoregulation [21]. Subjacent to the periostracum lies the prismatic layer—a mineralized layer composed of aragonite or calcite crystals developed in a columnar or prismatic structure perpendicularly to the shell surface [22,23]. Beneath this layer conferring the shell’s primary strength, rigidity, protection, and weight reduction, the nacreous layer, also called the mother-of-pearl, includes minute, thin overlapping sheets of aragonite crystals that are indistinguishable at low magnification [21]. Embedded in an organic matrix of conchiolin, this smooth, shiny layer serves as the innermost layer and provides the shell with both flexibility and strength [24].

At the microscale level, the crossed-lamellar structure and the prismatic structure are the most common types of microstructures [25]. The former structure consists of multiple thin layers (lamellae) of crystal fibers, arranged in successive layers and at different angles in relation to each other (generally 60° or 90°), interspersing with thin organic layers [25]. Residing within the middle to inner layers, this type of microstructure provides internal support and resilience, ensuring that any cracks that penetrate the outer layer are stopped or deflected before they can cause significant damage. On the other hand, the core elements of the latter structure, i.e., column-shaped crystals, are positioned at right angles to the surface [25,26]. Commonly found in the outer layer of the shell, this configuration is at the base of the shell and gives it hardness and resistance to compressive forces, protecting against direct impacts and environmental wear [26]. With such interlocked and complex hierarchical configuration, the mollusk shell allows for the dissipation of energy across multiple scales, absorbing impacts and resisting fractures [2,23]. This is especially important in bone graft materials, where the fusion of the graft with existing bone and the ability to withstand mechanical loads are critical for success.

## 3. Shell-Derived Biomaterials in Dental Applications

The core features of biomaterials used for dental applications are osteoconductivity and osteoinductivity [2,3]. Osteoconductive materials function as a scaffold on which cells attach, proliferate, and form new bone layers. As an example, new bone formation induced by substances such as Bio-Oss and Fisiograft is effective in clinical cases of periodontal defects and traumatic injuries, revealing their osteoconductive potential [27,28]. Other materials such as synthetic hydroxyapatite (HA), biphasic calcium phosphate (BCP), and beta-tricalcium phosphate (β-TCP) share these osteoconductive properties [29,30]. To create effective osteoconductive scaffolds, a pore size of 100–500 micrometers (μm) is recommended as this range provides a balance between facilitating cell migration, nutrient/waste exchange, and vascularization [31].

On the other hand, osteoinductive materials promote the differentiation of progenitor cells into osteoblasts and hence new bone formation, even in non-bony environments [32]. Thus, a demineralized bone matrix (DBM) is inherently osteoinductive because it can induce de novo bone formation [27,33]. In the context of optimal osteoconductive scaffolds, a porosity range between 50% and 90% is generally recommended [34]. Besides the combination of osteoconductive scaffolds with osteoinductive factors or surface treatments like argon plasma treatment (PAT) [28,35], the porous structure of materials plays an important role in bone regeneration. In fact, porous HA coatings can promote early bone ingrowth and fixation compared to dense coatings [36]. Therefore, dental materials should ideally possess both osteoconductive and osteoinductive qualities for effective bone regeneration [31].

Recent research has emphasized the importance of using appropriate bone grafts, either as autologous or synthetic bone substitutes, given the unique embryonic origin of the jaw bone from the neural crest—unlike most other bones in the body, originating from the mesoderm during embryonic development [37]. The main advantages of synthetic bone substitutes are their high availability, customizable properties, consistent quality, and reduced risks of disease transmission and donor site morbidity, hence eliminating the need for invasive harvesting from the patient [30,38]. As a synthetic substitute, hydroxyapatite shares similar characteristics with bone apatite (e.g., hexagonal structure, stoichiometric Ca/P ratio of 1.67) and possesses high thermodynamic stability under physiological conditions compared to other forms of calcium phosphates [39]. From a dental perspective, nanosized hydroxyapatite (nano-HA) is of particular interest among different HA structures due to its similarity in size, crystallography, and chemical composition with human hard tissues [40]. Importantly, the size of synthesized nano-HA crystals ranges from 20 to 80 nanometers (nm) [41]. The size of natural HA from human teeth and bones corresponds to this range [30]. This similarity is crucial for biomaterials applicable to dentistry since materials mimicking the natural size of bone and tooth crystals are likely to be more biocompatible and effective in promoting natural processes like bone integration and dental repair [42].

Several biomaterials recovered from molluskan shells possess substantial relevance for dental applications. A high-profile compound is calcium carbonate, present in molluskan shells as calcite or aragonite. These two substances with the same chemical composition but different crystal structures and properties (i.e., polymorphs) have wide applicability as starting materials in the synthesis of hydroxyapatite [1,2,29,30,42]. Other biomaterials derived from mollusk shells with potential applicability in dentistry are chitin (and its deacetylated derivative chitosan) found in their organic matrix [11,12,43]; nacre, typically found at the inner shell layer and composed of alternating layers of aragonite and a protein matrix [21]; and conchiolin, a protein-based material found in the outer layer of some mollusk shells [44,45].

With a long-standing tradition, the therapeutic use of mollusk shells is intricately linked to the cultural heritage of various communities. Thus, powdered shells were applied in traditional Chinese medicine to treat indigestion and bone disorders. In Ayurvedic medicine, molluskan shells were also a part of treatments for improving bone health and healing fractures [46]. In the contemporary era, these calcareous exoskeletons were used, among other purposes, for the preparation of toothpaste. An example is a prescription obtained from the 19th-century Spanish medical press about the usefulness and efficacy of tooth powders consisting of medicated soap, magnesium carbonate, pulverized cuttlebone, and essence of mint [46].

Importantly, the first definite proof of osteointegrated dental implant treatment in human history comes from the Mayan civilization and involves the use of snail shells [47]. Discovered by Dr. Wilson Popenoe and his wife Dorothy at Playa de los Muertos (Honduras), this mandible fragment from a 20-year-old woman includes three cuneiform shell pieces used to replace the three lower incisors [48]. Radiographic imaging demonstrated their integration into the dental bone, accompanied by the formation of new alveolar housing cavities [49]. Moreover, there was clear evidence that these implants were in service for several years [47,50]. This very early use of mollusk shells as dental implants is one of the first known instances of biocompatible materials being used to restore oral function, predating modern dental techniques by millennia. The success of these ancient implants highlights the inherent suitability of mollusk shells for use in dental applications—a suitability that modern science is now beginning to explore and validate.

### 3.1. Molluskan Shells as Sources of Hydroxyapatite

**Figure 2:**
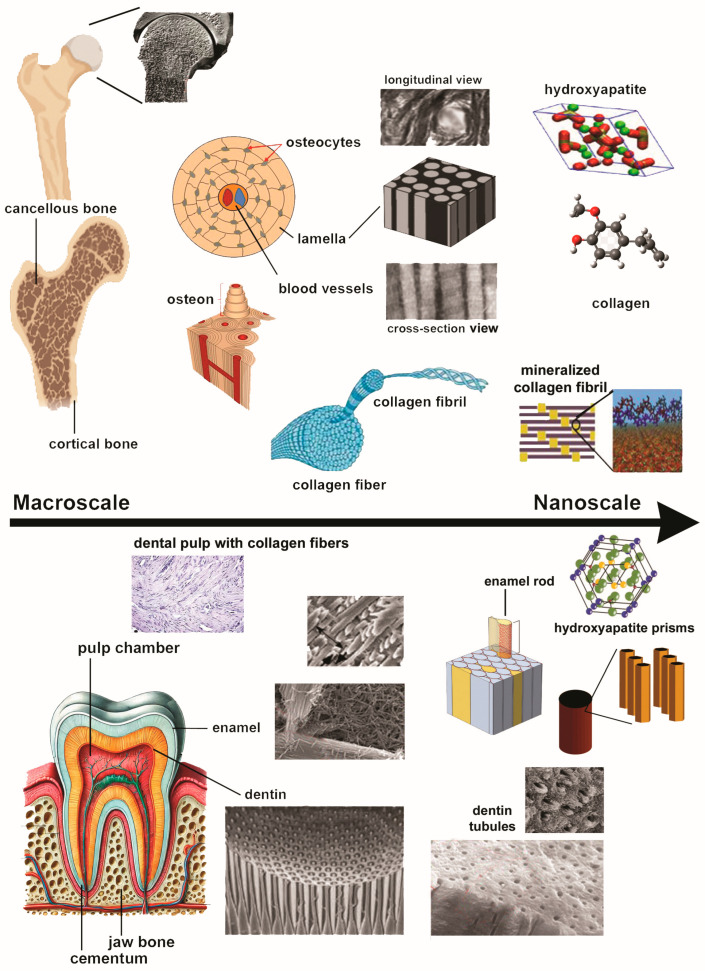
Hierarchical structure of bone (**upper** figure) and tooth (**lower** figure).

A natural form of calcium apatite with a typical lattice structure of [Ca(PO_4_)_6_(OH)_2_], hydroxyapatite is found in human bones and teeth [51]. It makes up to 65–70% of bone mass and is interspersed with type-I collagen at the nanoscale level, with nanosized particles dispersed throughout the collagen network [52]. This composite structure provides essential support for tissue stability and function, acting as a precursor to various biological mineralized tissues, e.g., tendons, skin, bones, and teeth [53]. In teeth, hydroxyapatite constitutes 70–80% of dentin, the inner layer of teeth, and enamel, the outer layer of the teeth. Unlike dentin, enamel, the hardest substance in the body, contains large HA crystals but no collagen. Instead, amelogenins and enamelins, the key proteins of enamel, provide the framework for mineralization, giving enamel its semitranslucent appearance [54]. Figure 2 illustrates a brief overview of the hierarchical structure of teeth and bones.

Given its distinctive properties that promote angiogenesis and accelerate bone healing, hydroxyapatite has become an indispensable material in modern dentistry [52,55]. More precisely, it promotes dentin remineralization during early carious processes by mediating the direct replacement of lost minerals and ion transport to the collagen network. Moreover, this calcium phosphate mineral can be applied as an implant coating, facilitating bone-to-implant contact, enhancing bone adhesion, and providing bacteriostatic benefits [40]. Nanoscale HA is especially noteworthy in the context of dental applications; it has higher solubility, surface energy, and bioactivity compared to hydroxyapatite, while sharing a similar structure with dental apatite [52].

Mollusk shells are a sustainable source of natural hydroxyapatite with remarkable osteoconductive properties [56]. As a result, HA synthesis from snails, bivalves, and cuttlefish has been extensively studied, revealing significant potential in many biomedical applications [2]. Although not naturally encountered in molluskan shells, hydroxyapatite can be recovered from these calcified exoskeletons via chemical processing [57]. In fact, the gastropod, bivalve, and cephalopod shells provide a low-cost and environmentally friendly path for HA production. As a result, hydroxyapatite synthesis using this avenue has therefore attracted growing research interest during recent decades [1,2], as evidenced by numerous studies on this topic (see Table 1 below).

Marine mollusks are generally preferred for biomedical applications. A major reason is that their shells tend to display higher calcium content and purity, e.g., 93.83% in the Asian green mussel, *Perna viridis* (Linaeus, 1758), versus 81.83% in the Giant African snail, *Achatina fulica* (Férussac, 1821) [58,59]. In addition, aquatic species and especially the inhabitants of oceanic environments have developed shells with superior mechanical properties, such as increased strength and resilience, to endure strong currents and provide defense against predators. [60,61]. Other advantages are the higher availability of raw materials, lower processing costs, and mature extraction techniques, leading to more cost-effective HA production [62]. In contrast, terrestrial mollusks generally require further processing to attain comparable levels of purity and strength. However, their use offers sustainability benefits, especially given their lower environmental impact and ease of controlled farming [63]. Nevertheless, both these sources of hydroxyapatite promote waste valorization and sustainability.

**Table 1:** Advantages and disadvantages of methodologies commonly used for recovering hydroxyapatite from molluskan shells.

Method	Advantages	Disadvantages	Reference
Thermal Treatment with Wet Precipitation	- High crystallinity - Tailorable particle size and morphology - Suitable for large-scale production	- High temperatures - Multiple steps, leading to increased complexity	[64,65,66,67,68,69,70,71,72,73,74,75,76,77]
Solid-State Reaction	- High purity - Simple process with fewer steps - Produces large quantities	- High temperatures - Limited control over particle size/morphology	[78]
Chemical Precipitation	- Relatively low-temperature process - Easy scalable	- Requires careful pH control - Possible formation of impurities if not properly controlled.	[79]
Hydrothermal Method	- Highly crystalline and pure HA - Can produce nano-sized particles - Environmentally friendly.	- Requires specialized high-pressure equipment - Longer reaction time	[80,81,82,83,84,85,86]
Sol–Gel Method	- -Produces highly pure and homogenous HA - Good control over HA composition and structure	- Complex preparation process - Requires precise control over synthesis parameters.	[87,88,89]

#### 3.1.1. Methods for Hydroxyapatite Synthesis from Molluskan Shells

Several methods have been frequently applied to recover HA from molluskan shells, especially dry methods, wet chemical processing, and mechanochemical reactions. Table 1 provides a concise summary of these methodologies. Microwave-assisted extraction was not included because it primarily differs from other methods by the use of microwave energy to accelerate the reaction process. The methods mentioned above (see Table 1) allowed scientists to obtain different compounds in terms of particle size, shape, and chemical composition [90]. The standard procedure begins with shell preparation, involving cleaning, crushing, and calcination to transform calcium carbonate into calcium oxide. The latter compound reacts with a phosphate source (phosphoric acid or ammonium phosphate), usually in the presence of water or under hydrothermal conditions, to form hydroxyapatite. The material is then subject to sintering—a process by which the powdered material is compacted and heated at a temperature below its melting point but high enough to allow the bonding of particles via diffusion processes [91]. This process, designed to enhance density, mechanical properties, and phase purity, is essential for producing dense and mechanically robust HA materials [5], but it is not always required in applications where mechanical strength is not the primary concern, e.g., in dentistry, for bioactive implant coatings or remineralization purposes.

However, the aforementioned stages can differ slightly based on the specific method employed. Thus, thermal decomposition involves calcining pre-cleaned shells at elevated temperatures to transform calcium carbonate into calcium oxide. The resulting calcium hydroxide is phosphatized post-hydration with a phosphate precursor to generate HA, which is dried and sintered (if needed) to increase crystallinity [92]. In contrast, chemical precipitation dissolves the shell material in an acid, typically hydrochloric acid, to produce a calcium ion solution, which then reacts with a phosphate solution under controlled pH conditions to precipitate hydroxyapatite. After aging to ensure complete precipitation, the mixture is filtered out, washed to remove impurities, dried, and then calcined (optional) at moderate temperatures to increase crystallinity [93]. Combining mechanical and chemical processes, the mechanochemical method is based on milling dried shells into a fine powder, which is mixed with a phosphate source and subjected to high-energy milling at a controlled temperature and for a specific time frame [94].

Other scientists converted molluskan shells into hydroxyapatite using the sol–gel reaction route; the shell powder was dissolved in an acidic solution and mixed with a phosphate precursor before allowing the formed gel (sol) to age and slowly dry at moderate temperatures [88]. Another method, the solid-state reaction, involves mixing shell powder with a phosphate source and heating the mixture in a furnace at high temperatures to facilitate the reaction [78]. It is also possible to use hydrothermal synthesis or microwave-assisted synthesis. The former method requires that clean shell powder is mixed with a source of phosphate in an aqueous solution at high temperatures and pressure for several hours to promote hydroxyapatite formation. The key step of the latter technique is to subject the mixture resulting from the acid digestion of shells and a phosphate source to microwave heating, which accelerates HA synthesis [95].

Reaction conditions exert a strong impact on the yield/quality of mollusk-derived hydroxyapatite. Temperature is crucial as it impacts reaction kinetics, crystal structure, material properties, and the overall stability of the final product [79]. Research findings support that initial calcination temperatures can be quite high (up to 800–1000 °C), whereas the reaction temperatures for most of the aforementioned paths (see Table 1) generally range between 80 and 250 °C [5,96]. For example, the optimal temperature range for obtaining high-purity mollusk shell-derived HA via the hydrothermal approach generally lies between 70 °C and 160 °C [80,85,97]. However, the selection of optimal ranges is shaped by the specific requirements for HA crystallinity, size, and purity. Thus, Fitriyana et al. (2023) investigated the effect of different reaction temperatures (120, 140, and 160 °C) on nano-HA produced from green mussel shells using a low-temperature hydrothermal approach. The highest quality of nano-HA was found at 160 °C [80]. Using the same mussel species and extraction method, Pratiwi et al. (2015) extracted hydroxyapatite at temperatures of 70, 80, and 90 °C after a preliminary calcination at 900 °C. The optimal nanoscale HA crystals occurred at 90 °C, for a stirring rate of 300 rpm, with higher reaction temperatures leading to smaller crystal sizes [85]. In contrast, the weight percentage and crystallite size increased with the reaction temperature [80].

The sintering temperature, influenced by the chosen synthesis method and desired material properties, plays a key role in defining the characteristics of the final product. As an example, it typically ranges from 800 to 1100 °C for direct thermal conversion. This approach is based on the direct reaction of CaO produced via calcination with phosphoric acid at elevated temperatures, without an intermediate dissolution and precipitation step like in the case of calcination with wet precipitation. At higher temperatures, HA crystallinity increases, but with the risk of obtaining undesired secondary phases [98]. In wet chemical precipitation, sintering temperatures are lower, generally varying between 600 and 900 °C. The desired application of the final material influences the choice of sintering temperature, with lower values being preferred for applications requiring preserved phase purity (e.g., orthodontic brackets, remineralization treatments) and higher temperatures (closer to 900 °C) being preferred for applications demanding high structural integrity (e.g., dental implants) [99]. In hydrothermal synthesis—with reactions occurring at lower initial temperatures (150 to 250 °C)—precipitated HA often undergoes sintering at 700 to 1000 °C to increase crystallinity and ensure a uniform phase composition [98]. Similarly, the optimal sintering temperature for the sol–gel method ranges from 800 to 1300 °C, with higher temperatures generally improving the compaction strength and reducing porosity [88]. One can hence expect that the size, density, and hardness of HA crystallites increase with the sintering temperature. However, balancing this parameter is essential to achieve the desired purity, crystallinity, and mechanical strength of the final product.

The synthesis of hydroxyapatite using liquid-phase extraction methods (e.g., wet chemical precipitation, sol–gel method) is a pH-dependent process. This stems from the fact that hydroxide ions are key players in stabilizing the crystal structure. The optimal range for the wet chemical precipitation is between 9 and 11, as demonstrated by Charlena et al. (2023) working with *Polymesoda placans* and Khiri et al. (2019) with *Anadara granosa* [64,100]. The ideal range for the sol–gel method is close, which is 8 to 10 [87,88]. This alkaline range promotes the dissolution of calcium, increases the availability of necessary ions, and promotes HA precipitation [28,101]. As pH changes can alter the quality of the final product due to the presence of undesired phases or impurities [28,101,102], a stable pH environment must be maintained during liquid-phase extraction methods [103].

The optimal reaction time also varies based on the synthesis method employed. Several study authors have reported that the hydrothermal method requires quite a long time (from 12 to 24 h) to ensure a complete reaction and formation of hydroxyapatite crystals [97,104,105]. Comparable durations, i.e., 6 to 48 h, are typically reported for the other methods of HA synthesis [62,78,87,88,104]. Microwave-assisted synthesis, by contrast, requires a shorter reaction time—often minutes—to produce hydroxyapatite [101]. Importantly, longer reaction times typically generate more crystalline and pure phases [86,106]. This reduces the presence of impurities and increases the structural integrity of the formed crystals as a result of a more complete nucleation and growth [79,98].

Aging time—a term used in the context of the sol–gel method—defines the time period required for the transition from sol (a colloidal suspension of particles) to gel (a semi-solid network) to allow gel maturation. Multiple factors, including the pH, processing conditions, and precursor concentration, can interact with this parameter [107]. In addition, longer aging times generate higher crystallinity, particle size, and phase purity but lower porosity [108]. Therefore, adjusting aging time and other related variables can affect the kinetics of HA formation and the final material characteristics [109,110].

Given its rich content in CaCO_3_, the shell material itself is the calcium precursor needed for the synthesis of hydroxyapatite from molluskan shells [111]. The phosphorus sources can vary depending on the desired chemical composition and structural properties of the final product. One of the most commonly used precursors is phosphoric acid. It was found that the reaction of phosphoric acid with calcium originating from the green mussel shells yielded hydroxyapatite with high crystallinity and structural integrity [59,88]. Similarly, the use of abalone shells as a source of calcium (with phosphoric acid being again selected as a phosphorus precursor) allowed the synthesis of HA and other calcium phosphates, such as β-tricalcium phosphate (β-TCP) [112]. Diammonium hydrogen phosphate (NH_4_)_2_HPO_4_ was also employed as a phosphorus source, particularly in the recovery of HA from mussel shells, generating nanoparticles with high crystallinity [59,69]. After a comprehensive compilation of the literature data, Venkatesan et al. (2018) reported that for cuttlefish bone-derived hydroxyapatite, H_3_PO_4_ is suitable to be used primarily in thermal treatment and wet precipitation method to generate porous particles, whereas (NH_4_)_2_HPO_4_ should be rather employed with the hydrothermal method to produce powders [113]. Scialla et al. (2020) also revealed that nano-HA particles with different features could be customized by changing thermal treatment conditions and phosphorus reagents; (NH_4_)_2_HPO_4_ yielded highly crystalline nanoparticles, whereas H_3_PO_4_ led to smaller particles with higher surface areas [92]. Overall, these data favor the use of phosphoric acid and diammonium hydrogen phosphate as the primary phosphorus precursors in the synthesis of HA from mollusk shells.

Xue et al. (2018) demonstrated that the porosity and mechanical strength of hydroxyapatite scaffolds can be modulated by controlling the soaking time and absorbed slurry composition. These scaffolds were derived from oyster shell powder through hydrothermal synthesis [114]. Charlena et al. (2015) recovered HA from the shells of the rice field snail *Bellamya javanica* (von dem Busch, 1844) via a wet method and investigated the impact of 4% and 6% chitosan on hydroxyapatite porosity. The pore size remained the same, but in vitro testing revealed an enhanced HA bioactivity [73]. Qiqing et al. (2017) found that hydroxyapatite morphology can be adjusted by the concentration of the organic template (sodium lauroyl sarcosine and cetyl trimethylammonium bromide) when synthesizing nano-HA via a chemical precipitation method based on shell powder and phosphate as precursors [115]. Furthermore, Cestari et al. (2021) showed that HA-related crystalline phases depend on the Ca/P ratio and the presence of different ionic species (e.g., Mg^2+^, Sr^2+^) [116].

Thermal treatment combined with wet precipitation is one of the most common strategies used to produce hydroxyapatite from mollusk shells (see Table 1); it is an efficient, cost-effective, and easily scalable method for obtaining HA particles with controlled stoichiometry, phase composition, and morphology [117,118,119]. Using this approach, Singh and Purohit (2011) produced powders from the shell of the brown garden snail, *Cornu aspersum* (Müller, 1774). The final product revealed appropriate physicochemical and biological attributes to promote bone formation, as evidenced via XRD, DTA/TGA, FTIR, and SEM analysis and soaking in a simulated body fluid (SBF)—a biomimetic method that mimics the natural processes of bone formation [77]. Puspitawati et al. (2023) obtained mussel-derived hydroxyapatite at different temperatures (700, 750, 800, 850, and 900 °C) and H_3_PO_4_ concentrations (0.4, 0.6, 0.8, 1, and 1.2 M). The best results were obtained at 900 °C and a 1.2 M phosphoric acid, yielding a product with 100% purity [69]. Charlena et al. (2023) recovered HA from *Polymesoda (Neocyrena) placens* (Hanley, 1844) with an equivalent technique, but used diammonium hydrogen phosphate as a phosphate source. The optimal conditions for producing HA of high crystallinity (>90%) were a sintering temperature of 1000 °C and a pH of 10 to 11. These values were determined after experimenting with various sintering temperatures (600, 800, 1000, and 1100 °C) and pH values (9, 10, and 11). The so-produced material displayed uniform granule particles with particle sizes of 0.3–1.6 µm [64]. This particle size range is suitable for dental applications since it provides a high surface area, balanced resorption, and mechanical strength [120].

Similar procedures using thermal treatment in conjunction with wet precipitation were applied to many species of bivalves and gastropods, e.g., *Corbiculacea* sp. [69]; donkey’s-ear abalone, *Haliotis asinina* (Linnaeus 1758) [121]; or the ark clam shell *Anadara granosa* (Linnaues, 1758) [70]. However, alternative methods (see Table 1) have been equally successful at recovering hydroxyapatite from mollusk shells. For example, Koonawoot et al. (2011) used the solid-state reaction and obtained powders displaying phase transformation from calcium phosphate to hydroxyapatite and incorporating both phosphate and hydroxyl groups, with potential use in biomedical applications [78]. Using the hydrothermal method and gastropod shells, Zuliantoni et al. (2022) extracted hydroxyapatite with particle sizes ranging from 26.9 μm to 322 μm. This wide range of particle sizes points to potential variability in the properties of HA obtained from snail shells. The corresponding crystal structures were rhombohedral and orthorhombic, as revealed by XRD analysis, whereas the synthesized HA contained both phosphate and hydroxyl groups, as found via FTR analysis [81]. These are key qualities of biomaterials that support dental bone formation, regeneration, and integration [1,3]. Furthermore, experimental data indicate that sol–gel synthesis and hydrothermal synthesis may be more effective for obtaining nano-HA with superior crystallinity, phase purity, and porosity from mollusk shells [98,122]. These features are distinctly advantageous for high-end biomedical applications in dentistry, such as bioactive coatings for titanium implants or advanced bone tissue engineering scaffolds [40,123,124,125].

Santosh and Prabu (2013) synthesized nano-HA from seashells using a wet chemical reaction and phosphoric acid as a phosphorus precursor, both with and without microwave irradiation. The former approach yielded particles of smaller size (68 nm vs. 101 nm) but with a higher aspect ratio (5.98 vs. 3.37). The increased aspect ratio indicates that the particles produced with microwave irradiation are more elongated and possess a larger surface area relative to their volume, leading to improved mechanical strength, osteoconductivity, and bioactivity [75]. Ramli et al. (2012) examined the effect of microwave irradiation on mussel-derived hydroxyapatite. The modulation of exposure time yielded nanocrystallites of different sizes (10–55 nm) and morphologies, with the size increasing with the irradiation duration [95]. Shavandi et al. (2014) converted waste green mussel shells into nano-HA using a rapid microwave irradiation method (900 °C, 30 min) as a faster alternative to conventional hydrothermal treatment. The as-produced, nanorod-shaped, faceted particles were less than 100 nm, exhibiting high purity (similar to commercial products) and remarkable heat stability at 1000 °C [126]. These data suggest that microwave-assisted synthesis can provide significant advantages in reaction speed, energy efficiency, and control over material properties [127,128].

#### 3.1.2. Biocompatibility and Applications of Mollusk-Derived Hydroxyapatite

While the aforementioned investigations demonstrate that mollusk-derived HA can be used for dental bone regeneration, its utility extends far beyond dental applications [129]. A promising area is the coating of titanium implants, where hydroxyapatite can significantly improve the integration of implants within bone tissue. This coating technique leverages the remarkable properties of mollusk-derived HA to augment the performance and longevity of titanium-based medical devices. Thus, Dorcioman et al. (2023) synthesized thin films from seashell-derived hydroxyapatite. These biofilms were hydrophilic, with the measured values of contact angles (i.e., 15° to 18°) being beneficial for cell–matrix adhesion and migration. These biofilms also revealed high durability, with a bonding strength of 49 millipascals—above the standard required by ISO regulations for high-load implant coatings. In addition, these biological coatings showed low cellular toxicity (including for osteoblasts, fibroblasts, and epithelial cells), strong antimicrobial properties, and good mineralization capacity when immersed in biological fluids [130]. The most common methodologies used for extracting mollusk-derived HA, as well as their advantages and drawbacks, are shown in Table 2. 

Plasma spraying and electrophoretic deposition are the most commonly referenced techniques in the clinical literature for coating titanium implants with mollusk-derived HA. It was demonstrated that plasma spraying allows for the deposition of thick, rough coatings suitable for enhancing osseointegration [131]. A key factor affecting the properties of these coatings is the temperature used during HA synthesis. For example, Hussain et al. (2023) deposited (on titanium) powders obtained from Indian clam seashells over a 2 h period at temperatures from 700 up to 1000 °C. The best adhesion strength, microhardness, wear resistance, and crystallinity were seen in samples obtained at 900 °C [132]. On the other hand, electrophoretic deposition is known for its ability to provide precision in coating thickness. Kristianto et al. (2022) extracted hydroxyapatite from the shells of the donkey’s ear abalone, *Haliotis asinina* (Linnaues, 1758), using the precipitation method. This material was subjected to different versions of electrophoretic deposition dip coating to evaluate its potential as a material for the surface coating of the titanium alloy in bone implants. The most homogeneous and thick HA layers were observed at a direct current voltage of 50 V and a withdrawal speed of 0.1 mm/s; higher calcination temperatures and voltages resulted in denser coatings with fewer defects [68].

Several comparative studies have shown that mollusk shell-derived materials perform as effectively as, or even surpass, conventional materials in a range of medical applications. For example, Dhanaraj et al. (2020) compared the nanocrystaline hydroxyapatite obtained (through microwave irradiation) from calcium nitrate (Ca(NO_3_)_2_ × 4H_2_O) and the virgin murex, *Chicoreus virgineus* (Röding, 1798). The latter material revealed better rod-like morphology, crystallinity, size, shape, surface area, and antibacterial activity [133]. Similarly, Cestari et al. (2021) investigated nano-HA extracted from cuttlefish bones, mussel shells, chicken eggshells, and amorphous calcium carbonate. These nanoparticles were obtained using wet mechanosynthesis (milling time: 30 min or 4 h) and successive drying in an oven (120 or 150 °C), followed by consolidation via uniaxial pressing and sintering (800–1100 °C). These nano-HA materials were non-cytotoxic according to the results of the Lactate Dehydrogenase (LDH) assay on MRC5 cells. Good adhesion and proliferation was observed for the MG63 osteosarcoma cell line at day 1, 3, and 5 post-seeding for all materials, with the egg-derived nano-HA exhibiting the best cell adhesion pattern, followed closely by the mussel-derived material. In contrast, hydroxyapatite recovered from cuttlebone and amorphous calcium carbonate displayed round-shaped cells and poorer cell-to-cell interconnection [116]. These findings suggest that mussel shells are more suitable than cuttlefish bones for synthesizing nanophase hydroxyapatite for biomedical applications.

In addition to its good osteoconductive and osteoinductive properties, mollusk-derived nano-HA possesses notable antimicrobial properties. Ahmed et al. (2022) reported that nanoparticles obtained from *Atactodea glabrata* (Gmelin, 1791) via thermal treatment with wet precipitation display potent inhibitory activity against *Staphylococcus aureus*, *Candida albicans*, *Bacillus subtilis*, *Klebsiella pneumoniae*, and *Escherichia coli* compared to conventional antibiotics. The measured values for minimum inhibitory concentration (MIC) were as low as 0.97 µg/mL in the case of the first two species. In addition, these nanoparticles demonstrated strong antibiofilm activity against *S. aureus* and *B. subtilis* [66]. In contrast, Sidauruk et al. (2023) found only a relatively weak, yet detectable antibacterial activity against *E. coli* for nanosized HA obtained from the shells of unionid clams (*Pilsbryoconcha* sp.) despite using a similar synthesis approach [134]. It was also demonstrated that the incorporation of transition metal ions into mollusk-derived hydroxyapatite strongly inhibits the replication of *S. aureus* [133]. Furthermore, thin films obtained from seashell-derived HA are promising candidates for dental implant coatings. These films induced a 10- to 1000-fold reduction in the growth rate of *Escherichia coli*, *Escherichia faecalis*, and *C. albicans* after two days of contact, while maintaining low toxicity to various cell types, including osteoblasts, fibroblasts, and epithelial cells [130].

Finally, hydroxyapatite recovered from mollusk shells have emerged as a promising material for dentine remineralization [135]. Thus, Bhavan Ram et al. (2023) revealed that oyster shell-derived nano-HA is effective for treating dentin hypersensitivity. It induced significantly greater dentinal tubule occlusion, deeper penetration, and reduced dentin permeability after remineralization versus the untreated controls, regardless of whether a 15% proanthocyanidin pretreatment was applied or not [136]. Similarly, Sari et al. (2022) showed that gels based on nano-HA produced from the shells of the tropical abalone *Haliotis asinina* and carbomers (thickening agents in dental care products) can promote enamel remineralization while preserving cell viability. The effectiveness of these gels depended on carbomer concentrations, and the best results were identified for mixtures where 20% of the total weight consisted of carbomers [137]. Hickmah et al. (2019) also found that the application of nano-hydroxyapatite extracted from blood clam shells increased calcium enamel concentrations after extracoronal bleaching [138].

### 3.2. Mollusk-Derived Carbonated Hydroxyapatite

As a modified form of HA with carbonate ions replacing several phosphate groups in its crystalline structure, carbonated hydroxyapatite (CHA) mimics the composition of natural bone more closely, enhancing its bioactivity and resorption rates. A brief comparison of HA and CHA related to their composition, structure, and properties is given in Table 3. It can be inferred from these data that incorporating carbonate ions into carbonated hydroxyapatite decreases crystallinity and enhances solubility, thereby improving biocompatibility, biomineralization, bioactivity, and reabsorption [139,140,141,142,143]. The synthesis path affects the morphology, size distribution, mechanical properties, surface chemistry, purity, and biological properties of CHA powders [144,145]. For example, the use of the hydrothermal method, wet precipitation, and microwaved-assisted synthesis results in nanorod-shaped CHA, nanocrystaline powders, and microsized granules, respectively [146].

The selection of hydroxyapatite or carbonated hydroxyapatite in dentistry is based on specific clinical considerations. More precisely, HA might be favored for dental bone grafts where slow resorption is advantageous (e.g., ridge augmentation, maxillary sinus augmentation, socket preservation, horizontal bone defects), coatings for dental implants, and products for enamel remineralization; it behaves as a scaffold for bone growth due to its stable and strong structure. Given its enhanced bioactivity and ability to closely mimic natural bone, CHA, on the other hand, might be best suited for applications requiring faster bone regeneration and integration, such as bone grafts (e.g., bone defects in orthodontic treatments, minor ridge augmentation), periodontal regeneration (e.g., infrabony defects, guided bone regeneration), and bioactive coatings for dental implants [146]. On the other hand, carbonated hydroxyapatite demonstrates improved uniformity and capacity, especially when produced at the nanoscale [148]. Typically, in nanoscale form, it provides an increased surface area, facilitating uniform distribution and interaction with its surrounding environment. Nevertheless, nanosized apatites are favored for pharmaceutical delivery due to a higher surface area-to-volume ratio, while larger microsized apatites are mainly utilized for bone graft and implant coatings [149].

Several studies have shown the successful CHA synthesis from different molluskan sources, including oyster, abalone, and gastropod shells, and via diverse methods, such as (co)precipitation, thermal decomposition, hydrothermal treatment, and wet chemical deposition [150,151,152,153,154,155,156,157,158]. Research indicated that the chemical composition of nano-CHA derived from the shells of *Haliotis asinina* via wet precipitation depends on aging time—the period during which the precipitated carbonated hydroxyapatite is maintained in solution under controlled conditions before being filtered and dried. More precisely, the content of Ca^2+^ and PO_4_^3−^ ions increased, whereas crystallinity, the percentages of carbon (C) and carbonate (CO_3_^2−^), and the molar Ca/P ratio decreased with aging time [121,158]. A similar trend was observed in particle size and their degree of size/molecular mass non-uniformity (polydispersity), but the aspect ratio (length/width) was independent of the aging duration [121].

The typical sintering temperatures for carbonated hydroxyapatites are similar to that reported for hydroxyapatite, i.e., 700 to 1200 °C (see Section 3.1.1). The content of carbonate or water in CHA decreases, whereas the crystallinity and crystallite size increase with increasing sintering temperatures [159]. Sintering at lower temperatures may therefore retain more of the carbonate content, which is beneficial for applications requiring higher bioactivity and faster resorption rates. Higher sintering temperatures, by contrast, may enhance mechanical strength but could lead to carbonate loss [149,160].

In a study with the Pacific oyster, *Crassostrea gigas*, Almukaramah et al. (2020) reported that carbonate substitution in CHA reduces crystallinity and crystallite size [161], in line with theoretical expectations. Anggraini and Yusuf (2019) found a time-dependent effect of stirring duration on the crystal structure, functional group intensity, and morphology of carbonated hydroxyapatite derived from the Australian South Sea pearl oyster, *Pinctada maxima* (Jameson, 1901) [162]. Working with the same species and a precipitation method with a short aging time (15 min), Megawatti et al. (2023) produced both A-type and B-type CHA particles, depending on the synthesis temperature. A higher temperature was associated with increasing crystallinity and the predominance of the B-type CHA [163].

Ge et al. (2016) identified the pH as an important modulator of the morphology and the degree of carbonate substitution in seashell-derived CHA. Raising the pH increased the amount of carbonate-substitution and the prevalence of sheet-like shaped over needle-like shaped nanoparticles [154]. The application of the CKK-8 assay—a relevant test for dental bone regeneration [162]—revealed that CHA significantly improves bioactivity compared to controls, with needle-like particles displaying better bioactivity [164]. These outcomes are consistent with the findings of Anggraini et al. (2019), showing that pH exerts a strong influence on the shape and structure of carbonated hydroxyapatite powders obtained from the shells of the pearl oyster *Pinctada maxima*. Thus, higher pH levels result in smaller crystal sizes, higher carbonate content, and a lower Ca/P ratio [165].

Setyoko et al. (2023) demonstrated in silico that carbonated hydroxyapatite from cuttlefish shells could prevent orthodontic relapse by disrupting the link between RANK and RANKL and enhancing the expression of OPG and TGF-β, yielding improved bone remodeling and stability after orthodontic treatment [166]. Cahyati et al. (2024) synthesized oyster-derived B-type CHA via precipitation, which was then freeze-dried together with honeycomb and polyethylene oxide to obtain composite scaffolds. The final product displayed a small-scale pore structure (micropore) favorable to cell migration, making this material a promising candidate for bone tissue engineering [152].

Based on the aforementioned data, one can conclude that the strong points of CHA versus HA involve enhanced bioactivity and bone mimicry, high resorption rate, customizable synthesis, and increased surface area at the nanoscale. Its main limitations are lower mechanical strength, variable stability, and carbonate loss at high temperatures. Taking these differences into account, CHA and HA derived from mollusk shells have the potential for distinct applications within different areas of dentistry. Thus, carbonated hydroxyapatite appears to be more appropriate for cases requiring faster bone regeneration and resorption, e.g., scaffolding for periodontal regeneration, guided bone regeneration, rapidly integrating bioactive coatings for dental implants, enhanced bone grafts for minor alveolar defects in orthodontics, and socket preservation and ridge maintenance following tooth extraction. It also serves as a promising material for bone tissue engineering and drug delivery systems for dental applications. On the other hand, it is better suited for applications requiring long-term stability and progressive bone growth (e.g., long-lasting coatings for load-bearing dental implants, including bone grafts for ridge augmentation and maxillary sinus elevation, socket preservation following extraction in anticipation of implants, enamel remineralization and restoration in dental therapies, and orthodontic anchorage sites for bone regeneration in complex cases.

## 4. (Pre)clinical Trials

No human clinical trial has investigated the applicability of mollusk shell-derived HA (and derivatives) in dentistry. There is, however, an indication that these materials are promising dental bone substitutes. Thus, Ryu et al. (2003) demonstrated that HA powders prepared from oyster shells and phosphoric acid increase bone density in humans over three months [167]. In vivo animal studies have already shown that seashell-derived hydroxyapatite is effective for dental bone regeneration [72]. Taqa et al. (2023) incorporated 1% gold in seashell-prepared nano-HA and tested in vivo its effectiveness in repairing bone defects in mandibular rabbits. Nano-HA repaired and replaced lost bone without causing inflammatory side effects irrespective of the gold addition [65].

Compelling evidence also supports the use of hydroxyapatite from other sources in bone regeneration. For example, Canullo et al. (2016) used nanocrystalline Mg-enriched HA to preserve the alveolar socket and identified its full regeneration at 12 months, though with significant material resorption [168]. In a separate study, Canullo et al. (2012) used nano-HA for sinus grafting, achieving a radiographic vertical height of the grafted sinus of 13.75 mm and a 97% success rate for implants after 24 months [169]. Both these studies used synthetic hydroxyapatite. Moreover, Kattimani et al. (2019) employed eggshell-derived nano-HA as a graft in third molar post-extractive sockets. Compared to controls, patients treated with nano-HA showed an enhancement at early bone modulation phases, i.e., 83.33% versus 50% of trabecular bone [170].

It is also worth mentioning that preliminary testing has been already conducted on other marine biomaterials. Vickers et al. (2021) used coralline HA as a substitute graft in a 66-year-old woman with a large non-contained mandibular defect caused by an odontogenic cyst. At eight months, post-operative imaging revealed a well-integrated graft and a completely filled mandibular defect [171]. However, molluskan shells with a nacre-based structure (e.g., from oysters, abalones, trochid snails) are known to possess better biocompatibility and osteoinductive properties compared to coralline materials because of their organic matrix, which enhances cellular attachment and proliferation [172]. Their structural features (e.g., the crossed-lamellar architecture) provide superior mechanical properties, such as higher fracture toughness or flexibility, thereby making them more appropriate for load-bearing applications [1]. These biomaterial sources can be processed to enhance their hardness and exhibit good cytocompatibility, along with some antifungal properties, which are attributed to their hydrophilic and negatively charged surfaces. Furthermore, oyster shell-derived bone substitutes promote bone formation earlier than traditional ones and, in turn, result in faster healing [173].

## 5. Conclusions

- With a hierarchical structure and compositional similarities with human bone and teeth, mollusk shells display strength, flexibility, resilience, and a strong potential for integration into the human tissues.
- Composed primarily of calcium carbonate as aragonite or calcite, these exoskeletons can be processed into different compounds of dental interest, including hydroxyapatite (HA)—a biocompatible, bioactive, osteoconductive, and osteoinductive material.
- Mollusk shell-derived HA shows great promise in oral rehabilitation, particularly as a cost-effective alternative to synthetic bone substitutes; it is low-impact, abundant, sustainable, and customizable into nanosized particles with enhanced bioactivity.
- Given their efficiency, scalability, and ability to produce highly crystalline and pure materials, thermal treatment coupled with wet chemical precipitation and hydrothermal synthesis are the most common methods used for recovering HA from mollusk shells.
- Various reaction/processing conditions (e.g., temperature, pH, phosphate sources, sintering parameters) affect the size, purity, and crystallinity of the final product.
- Carbonated hydroxyapatite (CHA) possesses higher bioactivity, biocompatibility, and solubility but lower crystallinity compared to regular HA, facilitating faster integration with natural bone.

## 6. Future Perspectives for Research on Mollusk Shell-Derived Hydroxyapatite in Dental Applications

We show here that mollusk shell-derived hydroxyapatite and its derivatives offer promising potential as a biocompatible, sustainable, and cost-effective alternative to synthetic bone graft materials in dentistry. However, to fully harness its capabilities for clinical applications, ongoing research should address several key aspects of its production, functionalization, and application:

- Optimization of synthesis techniques: Focus on refining methods to achieve high purity, crystallinity, and bioactivity in HA for medical and dental use.
- Sustainable production processes: Investigate green synthesis and low-energy production methods to make HA manufacturing more environmentally friendly and cost-effective.
- Nanotechnology integration: Develop nano-HA with controlled particle size, shape, and surface characteristics to improve cell attachment and bone integration.
- Bioactive ion incorporation: Explore adding ions (e.g., magnesium, zinc) to HA to replicate natural bone composition and promote tissue integration.
- Mechanical properties enhancement: Modify sintering techniques to improve HA’s mechanical properties, aiming for better structural stability in load-bearing applications.
- Reaction condition optimization: Study how conditions like temperature, pH, and sintering parameters affect HA properties to tailor it for specific clinical applications.
- Cross-disciplinary collaboration: Promote partnerships across material science, biomedicine, and environmental engineering to advance HA clinical applicability.

## Figures and Tables

**Table 2:** Advantages and disadvantages of methodologies commonly used for recovering hydroxyapatite from mollusk shells.

Method Type	Specific Method	Brief Description	Reference
In Vitro Cell Culture	Cell Viability and Proliferation Assays	Assays like MTT, Alamar Blue, and live/dead staining determine the viability and proliferation of osteoblasts on hydroxyapatite	[59]
	Osteoblast Differentiation Assays	Determines alkaline phosphatase activity, mineralization (e.g., Alizarin Red staining), and expression of osteogenic markers (e.g., Runx2, OCN, OPN)	[78]
	Cell Adhesion and Morphology	Employs SEM and immunofluorescence to observe cell attachment, spreading, and morphology on hydroxyapatite surface	[81]
In Vivo Animal Models	Implantation Studies	HA implants are inserted into bone defects or subcutaneous sites in animals to assess new bone formation and material integration	[128]
	Histological Analysis	Bone tissues around the implant are sectioned and stained (e.g., H&E, Masson’s Trichrome) to examine bone formation and bone-material interface	[98]
	Micro-Computed Tomography (Micro-CT)	Offers high-resolution 3D images on bone features (volume, density, architecture) around the hydroxyapatite implant	[59]
Mechanical Testing	Push-Out or Pull-Out Tests	Measures the force needed to dislodge the HA implant from the surrounding bone, reflecting the strength of bone-material integration	[78]
	Compression and Bending Tests	Evaluates the mechanical properties of the bone-HA composite	[81]
Biochemical Assays	Calcium and Phosphate Content	Measures mineral deposition on HA using techniques like inductively coupled plasma mass spectrometry (ICP-MS)	[98]
	Osteocalcin and Osteopontin Assays	Quantifies these bone-specific proteins in the tissue or culture medium to indicate osteogenic activity	[128]
Surface Characterization	X-Ray Diffraction (XRD)	Analyzes the crystalline structure of HA and its similarity to natural bone	[59]
	Fourier Transform Infrared Spectroscopy (FTIR)	Identifies chemical bonds and functional groups, revealing the presence of bone mineralization	[78]
	Energy-Dispersive X-Ray Spectroscopy (EDS)	Offers elemental composition data of HA and the newly formed bone	[81]
Surface and Structural Analysis	Atomic Force Microscopy (AFM)	Measures surface roughness and topography (with effect on cell adhesion and proliferation)	[59]
	Scanning Electron Microscopy (SEM)	Offers detailed images of the surface morphology and microstructure of HA, illustrating its interaction with bone cells	[98]
	Transmission Electron Microscopy (TEM)	Provides high-resolution images to analyze the fine structural details of HA and its integration with bone tissue	[128]
Biodegradability and Bioactivity Tests	In Vitro Degradation Studies	Assesses the rate at which HA degrades in simulated body fluid (SBF) or other physiological conditions	[78]
	Bioactivity Tests	Evaluates the formation of apatite on the HA surface when immersed in SBF, indicating the material’s ability to bond with natural bone	[81]

**Table 3:** Comparison of composition, structure, and properties of HA and CHA.

Characteristic	Hydroxyapatite	Carbonated Hydroxyapatite	
Chemical composition	- formula: Ca_10_(PO_4_)_6_(OH)_2_; - Ca^2+^: (PO_4_)^3−^ ratio = 1.67	- formula: Ca_10_(PO_4_)_6−x_(CO_3_)_x_ (OH)_2−x_, where x is the degree of carbonate substitution for hydroxide/phosphate - carbonate content 2–8%	[147]
Structure	- crystalline structure similar to natural bone mineral; - highly ordered and stable lattice structure; - hexagonal crystal system;	- modified structure occuring as A-type, with (CO_3_)^2−^ ions replacing OH^−^ ions, or B-type, with (CO_3_)^2−^ ions replacing (PO_4_)^3−^ ions - less ordered lattice, more similar to biological apatite found in bone	[146]
Properties	- high crystallinity and stability; - lower solubility in physiological conditions; - excellent biocompatibility and osteoconductivity.	- lower crystallinity and higher solubility - enhanced bioresorbability and biodegradability - better mimicry of natural bone mineral, which is naturally carbonated - potentially higher bioactivity and faster integration with natural bone	[147,148]
